# Distress following the COVID-19 Pandemic among Schools’ Stakeholders: Psychosocial Aspects and Communication

**DOI:** 10.3390/ijerph20064837

**Published:** 2023-03-09

**Authors:** Arielle Kaim, Shahar Lev-Ari, Bruria Adini

**Affiliations:** 1 Department of Emergency and Disaster Management, School of Public Health, Sackler Faculty of Medicine, Tel Aviv University, Tel Aviv 6139001, Israel; 2 Israel National Center for Trauma & Emergency Medicine Research, The Gertner Institute for Epidemiology and Health Policy Research, Sheba Medical Center, Tel Hashomer, Ramat-Gan 5266202, Israel; 3 ResWell Research Collaboration, School of Public Health, Tel Aviv University, Tel Aviv 6139001, Israel; 4 Department of Health Promotion, School of Public Health, Sackler Faculty of Medicine, Tel Aviv University, Tel Aviv 6139001, Israel

**Keywords:** COVID-19, distress, psychosocial aspects, school stakeholders, communication

## Abstract

In response to the COVID-19 pandemic, many governments ordered school closures as a containment measure, with Israel being among over 100 countries to do so. This resulted in the abrupt shift to online and remote education for many students. Despite attempts to minimize the effects of disrupted education and create a dynamic virtual learning environment, the literature highlights various challenges including lack of communication with implications of distress faced by key stakeholders (students and their parents, teachers, and principals). In this cross-sectional study, we assess the perceived levels of communication and psychosocial aspects during both distance and frontal learning, as well as the long-term impacts (following over two and a half years of an ongoing pandemic) on distress among the key stakeholders of the Israeli education system— high school students, parents, teachers, and principals. The study findings demonstrate severe implications of distance learning on communication and psychosocial aspects, with lingering long-term impacts on distress, among all stakeholders (particularly among students). This reveals the need for tailored capacity building and resilience intervention programs to be integrated in the long-term response to the current ongoing pandemic to improve well-being and reduce distress among the various stakeholders, with particular attention to those that are most vulnerable and were hit the hardest.

## 1. Introduction

As part of the coronavirus disease (COVID-19) pandemic containment measures, school closures were among the commonest governmental responses, with Israel joining over 100 countries in instituting this measure [1]. Governments had ordered institutions to cease face-to-face instruction for most of their students, requiring them to switch, almost overnight, to online teaching and remote education [2,3]. In the context of Israel, 15 March 2020, marked the official frontal closure of classrooms, where over 2.3 million pupils at all education levels were affected [4]. Since the initial closure of schools, the last (approaching) three years have seen a wax and wane trajectory in implemented measures as a response to the pandemic. Recurrent national or local lockdowns following additional waves of COVID-19 and the emergence of new and more infectious COVID-19 variants, resulted in additional school closures and distance learning. The estimated impact of these measures affects over 90 percent of the world’s school children (1.6 billion), according to UNESCO (2020). By the end of 2022, the weeks of school closure in some countries had extended to over 80 weeks [5]. The World Bank estimated that a school shutdown of 5 months could be worth 10 trillion USD in terms of learning losses [6], where on average, learning losses amount to 0.17 of a standard deviation, equivalent to roughly a one-half year’s worth of learning [7]. 

Beyond the impact on pupil academics, the closure of schools and the movement to distance learning limited the opportunities for peer-to-peer and student–teacher socialization, which is typically enhanced by in-person interactions [8]. Intricately tied, as contributing factors, to a lack of socialization and loneliness, are poorer mental health and higher levels of distress for both children and adults alike [9,10,11,12,13]. Despite efforts to mitigate and address the impact of disrupted education and create an engaging learning environment through virtual platforms, the literature has discussed various challenges that were encountered, presenting the conclusion that not all needs were fully met through this modality [12,13,14,15]. 

The implementation of distance learning, however, had much broader consequences on society than just on pupils. Pivotal impacts were observed among school staff and administration (principals), teachers, and parents [16,17,18]. Teachers and administrators were unable to carry out their teaching and facilitation process as usual and had to adapt to an online setting [19]. Many previously had no pedagogical experience in online teaching, where modalities needed to be adapted to digital platforms. These changes resulted in teachers reporting significant increases in workload, being burdened by the work, and socially isolated from fellow colleagues and students, resulting in high levels of distress [20,21,22]. Similarly, principals of schools also reported significant increases in workload [23]. In the context of the family, schools often provide safeguarding and supervision of children, allowing parents to work. When schools are closed, parents in some cases had to remain at home, with inevitable economic consequences, or they had to leave children unsupervised, similarly with implications for elevated parental mental distress [24]. As a result of COVID-19, school closures have shifted education from the classroom to the home, where parents had to fill an educational role as well (at least partially) [25]. It has previously been widely discussed in the scientific literature that communication between various stakeholders of school systems promotes improved well-being for all [26,27,28,29]. The findings of Lv et al., {2016} indicate that parent–teacher/ school communication plays an important moderating role in student emotional well-being and academic achievement, and Kuusimaki and Uusitalo-Malmivaara (2019) found that interactions between parents and teachers improve the well-being of teachers [26,27]. 

The global school closures due to COVID-19 have shown the fragility of education systems [30,31,32]. The uprooting and the reintegration of the global schooling system and its stakeholders because of the pandemic emphasize the need to ensure that school systems are resilient through all adversities. Functional resilience is defined as a system’s ability to resist, absorb, and respond to the shock of disturbances while maintaining its critical functions, and then recover to its original state or adapt to a new one [33,34]. Among the elements that functional resilience consists of are psychosocial aspects, communication between the varied stakeholders of the school system, digital literacy, pedagogic support, resources, and infrastructure (Kaim et al., under review). The ability to handle disruptions and maintain learning, teaching, and the support of the above activities is improved when all stakeholders are healthy (both physically and mentally). The capacities of education systems to respond to the crisis by delivering distance learning to support children, families, teachers, and administrators have been diverse and uneven [7]. The existing literature has predominantly focused on the adverse impacts of distance learning on the psychosocial aspects of all four key stakeholders, highlighting shared experiences of feelings such as isolation, disconnection, overwhelming, stress, and reduced motivation [8,9,10,11,12,13]. A lack of effective communication among these stakeholders has been a notable obstacle throughout the pandemic [12,13,14,15]. The long-term consequences of distance learning during the COVID-19 era remain largely uncertain, but there are indications that the negative effects may endure beyond the pandemic’s conclusion, including in the sphere of mental health [20,21,22,23,24,25]. The current study was targeted to cover this gap.

The aim of this study is to assess the perceived levels of communication and psychosocial aspects during both distance and frontal learning, as well as the long-term impacts (following over two and a half years of an ongoing pandemic) on distress among the key stakeholders of the education system—high school students, parents, teachers, and principals. As such, the study strives to contribute in its practicality, with direct and tangible policy implications for where the need is most apparent for capacity building and implementation of interventions targeted to improve the functional resilience of schools.

## 2. Materials and Methods

### 2.1. Study Design

Considering the importance of achieving an understanding of the long-term impact of COVID-19 on the stakeholders of the educational system, a cross-sectional study was conducted in October–November 2022, approximately two and half years after the initial closure of the frontal learning in the school system in Israel on 15 March 2022. A total sample of 1802 participants were recruited for this study, divided into the four key stakeholders of the education system: 10th–12th grade students (N = 1000), parents (N = 301), teachers (N = 449), and principals (N = 52) from 890 Israeli Jewish high schools. To partake in the study, the participants had to confirm their willingness to participate voluntarily in the study. The data was collected by the largest Israeli internet panel company which consists of over 140,000 panelists representing all demographic and geographic sectors (http://www.ipanel.co.il). This internet panel provides an online platform that adheres to the stringent standards of the European Society for Opinion and Marketing Research (ESOMAR). The data was collected anonymously, following approval of the Ethics Committee of the Tel Aviv University (number 0004549-1 from 13 February 2022) and the Ministry of Education (number 12379 from 28 April 2022). 

### 2.2. The Study Tool

The survey contained a brief introduction, which provided information on the background, objective, procedure, voluntary nature of participation, and declarations of anonymity and confidentiality. The four questionnaires were tailored to each of the stakeholders’ groups and consisted of six parts, based on items and indices that were developed specifically for this study, except for the perceived stress scale (PSS), which was based on a validated tool (PSS-4) [35] (See Appendix A). The number of items were not identical for the four tools, as to reflect the relevant relationships between stakeholders and the roles/functions that each stakeholder plays. The four questionnaires were validated by twenty content experts and pilot-tested on 25 individuals prior to their dissemination. The components of each of the questionnaires consisted of the following: Communication during distance learning, communication during frontal learning, psychosocial aspects during distance learning, psychosocial aspects in frontal learning, and PSS-4. 

#### 2.2.1. Communication during Distance Learning 

Communication during distance learning was measured by 3 items among students, 5 items among parents, and 7 items among teachers and principals. These components of the questionnaire encompassed attitudes of how well the communication was managed throughout the distance learning among all the stakeholders. Some of the questions were identical (though adapted to each specific population). The reliability of the scale was measured by Alpha Cronbach and results for each of the four stakeholders were (α = 0.833) among students, (α = 0.887) among parents (α = 0.861), among teachers and (α = 0.764), and among principals.

#### 2.2.2. Communication during Frontal Learning 

Communication during frontal learning was measured by 1 item among students, 2 items among parents, and 4 items among teachers and principals. The components of this questionnaire encompassed attitudes of how well the communication was managed throughout the frontal learning among all the stakeholders, whereas all stakeholders were asked to what extent they agree or disagree with the following sentences relating to when the school teaching was conducted through frontal learning, on a 5-point Likert scale, ranging from 1 = Disagree to a very great extent, to 5 = Agree to a very great extent. The reliability of the scale was measured by Alpha Cronbach and results for parents were (α = 0.612), (α = 0.812) among teachers, and (α = 0.797) among principals.

#### 2.2.3. Psychosocial Aspects during Distance Learning

Psychosocial aspects during distance learning were measured by 3 items among students and parents, and 5 items among teachers and principals. The components of this questionnaire encompassed attitudes towards distance learning among all the stakeholders, whereas they were asked to what extent they agree or disagree with the following sentences relating to when the school teaching was conducted through distance learning, on a 5-point Likert scale, ranging from 1 = Disagree to a very great extent, to 5 =Agree to a very great extent. The reliability of the scale was measured by Alpha Cronbach and results for each of the four stakeholders were (α = 0.819) among students, (α = 0.612) among parents, (α = 0.746) among teachers, and (α = 0.615) among principals. 

#### 2.2.4. Psychosocial Aspects during Frontal Learning

Psychosocial aspects during frontal learning were measured by 2 items among students and parents, and 5 items among teachers and principals. The components of this questionnaire encompassed characteristics of attitudes towards frontal learning among all the stakeholders where they were asked to what extent they agree or disagree with the following sentences relating to when the school teaching was conducted frontally, on a 5-point Likert scale, ranging from 1 = Disagree to a very great extent, to 5 =Agree to a very great extent. The reliability of the scale was measured by Alpha Cronbach as well as by Pearson correlation of items where only two items were used. The results for each of the four stakeholders are (r = 0.339) among students, (r = 0.357) among parents (α = 0.746), among teachers, and (α = 0.883) among principals.

#### 2.2.5. Perceived Stress Scale (PSS-4)

PSS-4 [35] was measured by four items among students, parents, teachers, and principals. The components of this questionnaire assess how often certain feelings and thoughts relate to the respondent in the past month for each stakeholder, on a 5-point Likert scale, ranging from 1 = never, to 5 =very often. The reliability of the scale was measured by Alpha Cronbach and results for each of the four stakeholders were (α = 0.668) among students, (α = 0.726) among parents, (α = 0.655) among teachers, and (α = 0.683) among principals.

#### 2.2.6. Demographics

Demographics for students were assessed by 11 items including gender, year of birth, place of residence, number of children living in the same home under the age of 18, number of dependents over the age of 18 living with you in the same home, religion, degree of religiosity, school type, school location, grade, and class size. For parents, teachers, and principals, demographics assessed included 12 items including gender, year of birth, place of residence, marital status, number of children under the age of 18 living with you at home, number of dependent adults living at home, education level, religion, level of religiosity, income level, school type, and location of the school. 

### 2.3. Statistical Analysis

Descriptive statistics were used to describe the participants’ demographic characteristics (frequency, mean, and standard deviation) of all four stakeholders. In addition, descriptive statistics were used to describe the characteristics of the schools sampled (%), and to determine the spread tendency and central tendency in the five indexes. A one-way ANOVA test was used to assess variability between stakeholders. A post hoc test (Bonferroni) was further conducted to enable the identification of the differences among the varied groups. Pearson correlation tests were conducted to analyze the associations between all variable indexes. All statistical analyses were performed using SPSS software version 28. *p*-values lower than 0.05 were considered to be statistically significant.

## 3. Results

### 3.1. Respondents and School Sample Characteristics

The sample of the study included 1802 participants, including 1000 students, 301 parents, 449 teachers, and 52 principals. Table 1 presents the demographic characteristics of all four stakeholders of the surveyed population. The average age of the students sampled was 16.7, with the majority (52.5%) being male. The average age of parents sampled was 48.0, with the majority being female (68.4%). The average age of teachers sampled was 41.6, with the majority being female (80.8%). Lastly, the average age of principals was 46.7, with the majority being female (55.8%). Among students, parents, and principals, the majority (54.4%, 59.1%, and 53.8%, respectively) considered themselves secular. Among teachers, the largest mass of the sampled population (43.0%) similarly considered themselves secular. The majority of parents, teachers, and principals (85.4%, 71.7%, and 88.6%, respectively) were in a relationship, with children. In addition, the majority of parents, teachers, and principals had received a bachelor’s degree and above. 

A total of 890 Jewish schools within Israel were sampled, where 67.1 % were state schools, and 32.9% were religious. The largest number of schools sampled geographically were from the central region of Israel (29.6%). Table 2 presents the characteristics of the schools sampled in this study. 

### 3.2. Mean Levels of Communication, Psychosocial Aspects and PSS 

The mean level for the communication index during distance learning among all the stakeholders was found to be 3.51 ± 0.88, as compared to 3.91 ± 0.87 during frontal learning. With regard to psychosocial aspects indices, during distance learning the mean level was 2.92 ± 1.04 as compared to frontal learning—3.72 ± 0.92. Furthermore, the mean PSS score among all participants was 3.46 ± 0.72. (See Table 3). 

### 3.3. Correlation between Variables

A positive, significant correlation was found between communication during distance learning and communication during frontal learning (r = 0.297), PSS (r = 0.264), psychosocial aspects during distance learning (r = 0.28), as well as psychosocial aspects during frontal learning (r = 0.211). Communication during frontal learning was found to be positively and significantly correlated with PSS (r = 0.285) and psychosocial aspects during frontal learning (r = 0.47). Lastly, PSS was found to be positively and significantly correlated with psychosocial aspects during both distance (r = 0.106) and frontal learning (r = 0.344). (See Table 4).

### 3.4. Differences by Stakeholder and Distance Versus Frontal Learning

**Figure 1:**
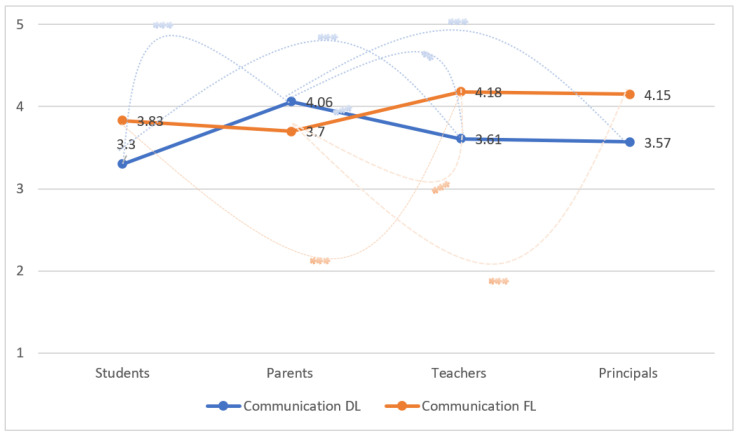
Communication Index (Distance Learning and Frontal Learning) according to the four stakeholders (Students, Parents, Teachers, Principals). Note: F = 72.54, *p* < 0.001 for differences for distance and frontal learning according to stakeholder as a result of repeated measure test. Statistical significance denoted as * (*p*-value less than 0.05 (*); *p*-value less than 0.01 (**), *p*-value less than 0.001 (***) according to Bonferroni’s multiple comparisons test.

Differences by stakeholders (students, parents, teachers, and principals) with respect to communication levels both during distance learning (DL) and frontal learning (FL) are displayed in Figure 1. During DL, students on average, display the lowest levels of the communication index (3.3), as compared to the other stakeholders, while parents display the highest levels (4.06). With regard to communication during FL, parents display the lowest mean levels of the index, while teachers indicate the highest levels. With regard to communication DL, significant differences are observed between students and parents (*p* < 0.001), students and teachers (*p* < 0.001), parents and principals (*p* = 0.001), and parents and teachers (*p* < 0.001). With respect to communication FL, significant differences are observed between students and teachers (*p* < 0.001), parents and principals (*p* < 0.01), and parents and teachers (*p* < 0.001). 

The results also suggest that each stakeholder during distance learning perceived the communication to be less effective as compared to frontal learning (students—3.3 DL versus 3.83 FL; teachers—3.61 DL versus 4.18 FL; principals—3.57 DL versus 4.15 FL), with the exception of parents (4.06 DL versus 3.7 FL). The differences were all significant according to the repeated measures test (F = 72.54, *p* < 0.001).

**Figure 2:**
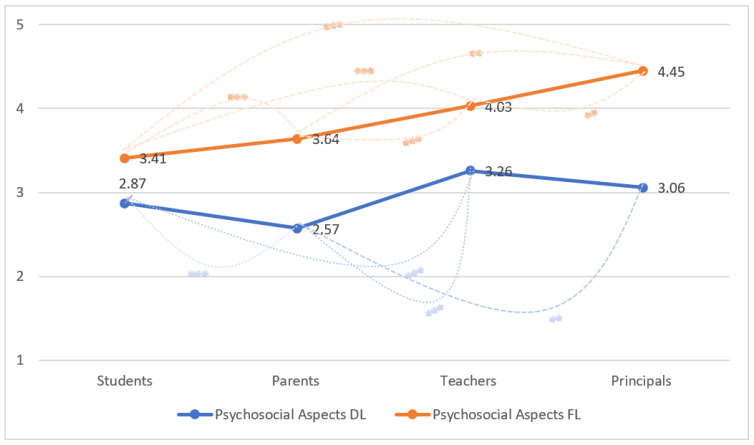
Psychosocial Aspects Index (Distance Learning and Frontal Learning) according to the four stakeholders (Students, Parents, Teachers, Principals) Note: F = 29.91, *p* < 0.001 for differences for distance and frontal learning according to stakeholder as a result of repeated measure test. Statistical significance denoted as * (*p*-value less than 0.05 (*); *p*-value less than 0.01 (**), *p*-value less than 0.001 (***) according to Bonferroni multiple comparisons test.

Differences by stakeholders (students, parents, teachers, and principals) with respect to psychosocial aspect levels, during both distance (DL) and frontal learning (FL) are displayed in Figure 2. During DL, parents on average display the lowest levels of the psychosocial aspects index (2.57 ± 1.04), as compared to the other stakeholders (students—2.87 ± 1.10, teachers—3.26 ± 0.83, principals—3.06 ± 0.64), with teachers displaying the highest levels. With regard to the psychosocial aspects during FL, students display the lowest mean levels of the index (3.41 ± 0.85), while principals indicate the highest levels (4.45 ± 0.1.14). With regard to psychosocial aspects during DL, significant differences are observed between students and parents (*p* < 0.001), students and teachers (*p* < 0.001), parents and teachers (*p* < 0.001), and principals and parents (*p* < 0.01). With respect to psychosocial aspects during FL, significant differences are observed between students and parents (*p* < 0.001), students and teachers (*p* < 0.001), students and principals (*p* < 0.001), parents and teachers (*p* < 0.001), parents and principals (*p* < 0.01), and principals and teachers (*p* < 0.01).

The results also suggest that each stakeholder during distance learning perceived psychosocial aspects to be less effective as compared to frontal learning (students—2.87 DL versus 3.41 FL, parents—2.57 versus 3.64, teachers—3.26 DL versus 4.03 FL, principals—3.06 DL versus 4.45 FL). The differences were all significant according to the repeated measures test (F = 29.91, *p* < 0.001).

**Figure 3:**
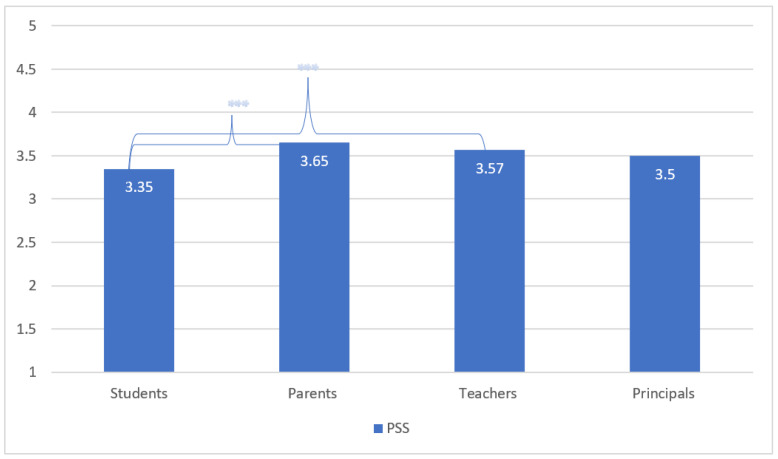
Perceived stress scale (PSS)-4 Index according to the four stakeholders (Students, Parents, Teachers, Principals). Note: 1 = Higher PSS, 5 = Lower PSS. Statistical significance denoted as * (*p*-value less than 0.05 (*); *p*-value less than 0.01 (**), *p*-value less than 0.001 (***) according to Bonferroni’s multiple comparisons test.

Lastly, the mean PSS-4 scores were 3.35 ± 0.72 for students, 3.65 ± 0.68 for parents, 3.57 ± 0.70 among teachers, and 3.50 ± 0.73 among principals. Significant differences were observed between students and parents (*p* < 0.001), as well as between students and teachers (*p* < 0.001). The lower the score, the higher the level of perceived stress, whereas the highest level of stress is exhibited among students (See Figure 3).

## 4. Discussion

The transition to distance learning for education systems posed a major challenge in the wake of the COVID-19 pandemic. With schools forced to close their doors, education leaders, teachers, students, and parents had to quickly pivot to a remote learning model in order to maintain educational continuity. The shift highlighted the need for functional resilience in the education system, specifically the system’s ability to adapt to unexpected disruptions and ensure the delivery of quality education. Through the prioritization of functional resilience, education systems and their stakeholders can not only overcome current adversities but be better prepared for future disruptions. Several of the key aspects identified as crucial to the functional resilience of a system are communication between relevant stakeholders and attitudes towards psychosocial aspects among stakeholders. 

The findings of this investigation demonstrate several interesting phenomena with regard to these two elements, as well as the long-term implications of the interaction between these aspects. First, students perceived levels of communication most critically during distance learning as compared to the other stakeholders, with parents expressing the highest levels. In contrast, when referring to perceived communication levels during frontal learning, parents indicate the lowest levels across all the stakeholders. This finding may be a reflection of the high home-based involvement of parents in supporting their children’s learning throughout the school closures, as compared to routine patterns of interaction in the home. The literature similarly suggests that parent involvement in children’s learning increased throughout distance learning, where the home-learning context improved relationships with teachers, with sentiments reciprocated by teachers [36,37]. The more critical attitudes of students toward distance learning match findings in the literature, where students have expressed decreased communication between them and their instructors and their increased feelings of isolation [38]. Teachers and principals report higher levels of communication during in-person learning, consistent with the challenges that education encountered while adapting to remote classes, with poor communication and low interaction as key hurdles faced [39,40].

Interestingly, psychosocial attitudes during distance learning were perceived lowest among parents regarding their children, indicating that parents’ perceptions of their children’s attitudes towards the learning experience during distance learning are worse than the children’s own perception. Parents consistently have been found in the context of health-related quality of life to rate their child’s health as worse than the child’s reporting, related to unresolved concerns regarding the influence of parental distress [41,42,43]. Furthermore, teachers expressed the best psychosocial attitudes toward distance learning (feeling more motivated and less burdened by the transition), as compared to the other stakeholders. Despite this, across all four stakeholders, the implications of distance learning on motivation, and attitudes towards the transition’s burdensome nature are clear, supporting previous findings [44,45,46]. 

The most pertinent implications of this study indicate the long-lasting impacts of distance learning, as reflected in the findings regarding the PSS-4 index, as the measurement assesses distress feelings and thoughts in the past month. The findings suggest that beyond the distance and frontal learning experiences, students exhibit the highest persisting levels of distress, potentially an indication of being hit hardest by frontal school disruptions, among the four stakeholder groups. Despite the fact that no previous assessment of PSS-4 was found in the literature on adolescents to assess differences with pre-pandemic PSS-4 scores, previous studies have discussed long-term lingering (extension into long-term even after the end of the pandemic) distress impacts among adults [47,48,49]. In the context of the current study, parents display among all the stakeholders, the lowest levels of distress according to the PSS-4 measurement, potentially indicating that this sample of the population, may be less vulnerable after their return to a work–balance routine and is thus able to better cope and bounce back following return to normalcy. Furthermore, a positive relationship between higher perceived levels of communication during distance learning, and higher perceived levels of psychosocial aspects was found in this study. In addition, a higher PSS index (in the context of this study, a lower level of stress), is correlated with higher perceived communication and psychosocial levels. The relationship between perceived levels of communication, psychosocial aspects, and perceived stress were found to be positively correlated in this study. Communication has previously been established as a protective factor from distress among school children, and improved well-being across the various stakeholders [26,29,50].

Interventions previously examined among high school students offer various solutions, including the integration of meditation apps which have been shown to improve perceived stress among adolescents, as well inquiry-based stress reduction among teachers [16,51]. Furthermore, the integration of tailored interventions in the context and in the midst of future scenarios among stakeholders should be considered in order to prevent lingering long-term repercussions and foster lasting resilience. At a broader level of functional resilience, the measures adapted by schools during the pandemic include the establishment and implementation of virtual learning infrastructures, provision of technical, pedagogic and psychosocial support, as well as provision of required resources [52].

The current study has shed light on the important role of psychosocial aspects and communication in the functional resilience of the education system. By examining the attitudes of four key stakeholders towards distance learning, the study has uncovered significant relationships between these attitudes and long-term distress. Additionally, this research work has pinpointed possible vulnerabilities that exist within the educational system, and where special dedicated attention may be warranted. To the best of our knowledge, few studies have been carried out to evaluate the impacts of COVID-19 distance learning on four key stakeholders, especially investigating the relationship between attitudes towards communications levels, psychosocial aspects, and long-term distress.

### Limitations

The study has several limitations. First, the study utilized an online panel to collect responses. While this methodology ensured a rapid turnover of information and provided a large sample of the Israeli Jewish population, the study conclusions are limited to individuals who have access to a source of internet and high computing skills. It should also be noted that though a very large sample of schools were included in the study, there is variability among schools concerning their technological, psychosocial and educational resources. Moreover, the study’s cross-sectional design limits its findings on attitudes towards distance learning, as they were not assessed during a school closure, but rather after. Furthermore, the findings of this study must be carefully considered, as generalizability and transferability to other societies may be limited; to support or refute the findings of this study, it is recommended that similar studies be carried out in additional schools in varied countries.

## 5. Conclusions

Our findings have valuable implications as we show the relationship between attitudes toward communications levels, psychosocial aspects, and long-term distress among four key stakeholders of the education system. The findings of the study reveal the need for tailored intervention programs to be integrated in the long-term response to the current ongoing pandemic to improve the well-being and reduce distress among the various stakeholders, with particular attention to those that are most vulnerable and were hit the hardest, (in the context of the current findings, the students). Future research should explore the longitudinal impacts of various interventions on changing long-term distress patterns among the various stakeholder participants. It is also recommended to expand the study beyond the Jewish schools context to include, for example, Arab or ultra-religious schools (in the Israeli context), as well as beyond Israeli borders.

## Figures and Tables

**Table 1:** Demographic characteristics according to the four stakeholder groups (Students, Parents, Teachers, and Principals).

	Total N = 1802	Students N = 1000	Parents N = 301	Teachers N = 449	Principals N = 52	*p*-Value
**Gender**						
Male	40.4	52.5	31.6	19.2	44.2	<0.001
Female	59.6	47.5	68.4	80.8	55.8	
**Age (Mean ± SD)**	29.4 ± 15.4	16. 7± 0.8	48.0 ± 5.1	41.6 ± 11.8	46.7 ± 8.9	<0.001 *
**Level of Religiosity**						
Secular	52.4	54.4	59.1	43.0	53.8	<0.001
Traditional	21.8	22.1	25.9	19.4	11.5	
Religious	25.9	23.5	15.0	37.6	34.7	
**Family Status**						
In a relationship, without children	5.1		0	8.7	3.8	<0.001 *
In a relationship, with children	77.9		85.4	71.7	88.6	
No relationship, no children	5.7		0	9.6	3.8	
No relationship, with children	11.3		14.6	10.0	3.8	
**Level of Education**						
Below high school school education	9.7		28.3	0	0	<0.001 *
Teacher seminar	3.2		0	5.0	3.9	
B.A.	46.9		44.6	50.2	29.4	
M.S or above	40.2		27.1	44.8	66.7	
**Level of Income**						
Below mean	40.0		36.9	44.9	14.5	<0.001 *
Mean	30.7		25.5	34.2	29.2	
Above mean	29.3		37.6	20.9	56.3	

Note: Data presented by percentage (%) or Mean ± SD. * Comparing only between parents, teachers, and principals. Missing data: Gender = 1, Level of education = 53, Level of religiosity = 5, Income level = 55.

**Table 2:** Characteristics of schools sampled (N = 890).

	Total N = 890	Percent (%)
**Type of school**		
State school	597	67.1
Religious school	293	32.9
**School region**		
North	115	13.1
Haifa	93	10.6
Tel Aviv	120	13.6
Center	261	29.6
Jerusalem	87	9.9
South	140	15.9
West Bank	65	7.4
**Number of students in school**		
100 and below	161	21.5
101–200	187	24.9
201–500	251	33.5
500 and above	151	20.1

**Table 3:** Spread tendency and central tendency of the index.

Index	Mean ± SD	Median	25th Percentile	75th Percentile
**Communication Distance Learning (DL)**	3.51 ± 0.88	2.40	1.86	3.0
**Communication Frontal Learning (FL)**	3.91 ± 0.87	2.00	1.25	3.00
**Psychosocial Aspects Distance Learning (DL)**	2.92 ± 1.04	3.00	2.33	3.80
**Psychosocial Aspects** **Frontal Learning (FL)**	3.72 ± 0.92	2.00	1.50	3.00
**Perceived stress scale (PSS)**	3.46 ± 0.72	3.5	3.00	4.00

Note: Minimum of every scale was 1 and maximum was 5.

**Table 4:** Correlation matrix between all variable indices.

	Communication: Distance Learning	Communication: Frontal Learning	Perceived Stress Scale	Psychosocial Aspects: Distance Learning	Psychosocial Aspects: Frontal Learning
**Communication: Distance Learning**	1				
**Communication: Frontal Learning**	0.297 **	1			
**Perceived Stress Scale**	0.264 **	0.285 **	1		
**Psychosocial Aspects: Distance Learning**	0.28 **	0.029	0.106 **	1	
**Psychosocial Aspects: Frontal Learning**	0.211 **	0.47 **	0.344 **	0.002	1

* Correlation is significant at the 0.05 level (2-tailed). ** Correlation is significant at the 0.01 level (2-tailed).

## Data Availability

The data collected in this study is not available in any public repository due to the regulations of the funder. The analyzed data will be made available to requesting researchers upon a reasonable request.

## Appendix A

Communication during distance learning: The items in the index for students included: one item which assessed “I had the opportunity to make personal contact with teachers and school staff”; one item “My teachers kept in touch with me on an ongoing basis”; and one item “The teachers monitored my progress to the extent required”. For parents, one item assessed “My child had the opportunity to make personal contact with the teachers and school staff; one item “My children’s teachers kept in touch with them on an ongoing basis”; one item “My children’s teachers kept in touch with me on an ongoing basis”; one item “Teachers monitored my child’s academic status and progress to the extent required”; and one item “A mutual update was made between me and my children’s teachers about their situation to a sufficient extent”. In both the teacher and principal questionnaire, one item assessed “I had the necessary means to maintain personal contact with students, parents, teachers and school management/ administration staff ” respectfully; one item “I maintained regular contact with the students”; one item “I maintained regular contact with the students’ parents”; one item “I kept in regular contact with the teachers”; one item “I kept in regular contact with the school’s management team”; one item “I/ Teachers was/were able to track the status and progress of the students to the extent required” respectfully; and one item “A mutual update was made between me/ the teachers and the parents of the students regarding the situation of the students to a sufficient extent”.

Communication during frontal learning: The item concerning communication during frontal learning for students assessed “In classroom learning, I maintain ongoing contact with my teachers”; For parents, one item assessed “In classroom learning, the teachers maintain ongoing contact with my children; and one item “In classroom learning, my children’s teachers keep in touch with me on an ongoing basis”. In both the teacher and principal questionnaire, one item assessed “I maintain ongoing contact with the students”; one item “I maintain ongoing contact with the students’ parents”; one item “I maintain regular contact with the teachers”; and one item “I maintain regular contact with the school’s management team”.

Psychosocial aspects during distance learning: The items in the index for students included one item which assessed “I enjoy distance learning”; one item “I am highly motivated to learn remotely”; and one item “Distance learning wears me out”. For parents, one item assessed “My child enjoys learning remotely”; one item “My child is highly motivated to learn remotely”; and one item “Distance learning has worn out my child”. In both the teacher and principal questionnaire, one item assessed “I am highly motivated to teach remotely/ manage while teaching remotely” respectfully; one item “Remote teaching/Managing the school remotely wears me out” respectfully; one item “I have confidence in my teaching/ management abilities and skills when teaching/ managing remotely” respectfully; one item “I receive sufficient support I need while teaching/ managing remotely to succeed in my work” respectfully. In addition, teachers were asked “I enjoy distance instruction”, while principals were asked “I benefit from school management while teaching remotely”. One item “wears me out/ wore out” was recoded to present the same directionality as the other questions.

Psychosocial aspects during frontal learning: The items in the index for students include one item which assessed “In classroom learning, I am highly motivated to learn” and one item “Learning in the classroom constitutes a heavy burden”. For parents, one item assessed “When learning in class, my child is highly motivated to learn”; and one item “Learning in the classroom constitutes a heavy burden”. In both the teacher and principal questionnaires, one item assessed “I am highly motivated to teach/manage” respectfully; and one item “Teaching/Managing frontally constitutes a heavy burden” respectfully. One item “heavy burden” was recoded to present the same directionality as the other questions.

Perceived stress scale-4: The items in the index include: “In the last month, how often have you felt out of control over the important things in your life”; “In the last month, how often have you felt confident in your ability to address your personal problems”; “In the last month, how often have you felt that things are developing according to your wishes?”; and “In the last month, how often have you felt that the difficulties are accumulating to such an extent that you cannot overcome them?” For analysis purposes, the answers of items 1 and 4 were recoded to present the same direction as the other two questions.
